# Proactive Suicide Prevention Online (PSPO): Machine Identification and Crisis Management for Chinese Social Media Users With Suicidal Thoughts and Behaviors

**DOI:** 10.2196/11705

**Published:** 2019-05-08

**Authors:** Xingyun Liu, Xiaoqian Liu, Jiumo Sun, Nancy Xiaonan Yu, Bingli Sun, Qing Li, Tingshao Zhu

**Affiliations:** 1 Institute of Psychology Chinese Academy of Sciences Beijing China; 2 Department of Psychology University of Chinese Academy of Sciences Beijing China; 3 Department of Social and Behavioural Sciences City University of Hong Kong Hong Kong China (Hong Kong); 4 Department of Computing The Hong Kong Polytechnic University Hong Kong China (Hong Kong)

**Keywords:** suicide identification, crisis management, machine learning, microblog direct message, social network, Chinese young people

## Abstract

**Background:**

Suicide is a great public health challenge. Two hundred million people attempt suicide in China annually. Existing suicide prevention programs require the help-seeking initiative of suicidal individuals, but many of them have a low motivation to seek the required help. We propose that a proactive and targeted suicide prevention strategy can prompt more people with suicidal thoughts and behaviors to seek help.

**Objective:**

The goal of the research was to test the feasibility and acceptability of Proactive Suicide Prevention Online (PSPO), a new approach based on social media that combines proactive identification of suicide-prone individuals with specialized crisis management.

**Methods:**

We first located a microblog group online. Their comments on a suicide note were analyzed by experts to provide a training set for the machine learning models for suicide identification. The best-performing model was used to automatically identify posts that suggested suicidal thoughts and behaviors. Next, a microblog direct message containing crisis management information, including measures that covered suicide-related issues, depression, help-seeking behavior and an acceptability test, was sent to users who had been identified by the model to be at risk of suicide. For those who replied to the message, trained counselors provided tailored crisis management. The Simplified Chinese Linguistic Inquiry and Word Count was also used to analyze the users’ psycholinguistic texts in 1-month time slots prior to and postconsultation.

**Results:**

A total of 27,007 comments made in April 2017 were analyzed. Among these, 2786 (10.32%) were classified as indicative of suicidal thoughts and behaviors. The performance of the detection model was good, with high precision (.86), recall (.78), F-measure (.86), and accuracy (.88). Between July 3, 2017, and July 3, 2018, we sent out a total of 24,727 direct messages to 12,486 social media users, and 5542 (44.39%) responded. Over one-third of the users who were contacted completed the questionnaires included in the direct message. Of the valid responses, 89.73% (1259/1403) reported suicidal ideation, but more than half (725/1403, 51.67%) reported that they had not sought help. The 9-Item Patient Health Questionnaire (PHQ-9) mean score was 17.40 (SD 5.98). More than two-thirds of the participants (968/1403, 69.00%) thought the PSPO approach was acceptable. Moreover, 2321 users replied to the direct message. In a comparison of the frequency of word usage in their microblog posts 1-month before and after the consultation, we found that the frequency of death-oriented words significantly declined while the frequency of future-oriented words significantly increased.

**Conclusions:**

The PSPO model is suitable for identifying populations that are at risk of suicide. When followed up with proactive crisis management, it may be a useful supplement to existing prevention programs because it has the potential to increase the accessibility of antisuicide information to people with suicidal thoughts and behaviors but a low motivation to seek help.

## Introduction

Approximately one million people die by suicide globally every year. Aside from being a great challenge to public health, suicide also causes significant economic losses and aggravates labor shortages. It is estimated that by 2020, approximately 1.53 million people will die from suicide annually, and the number of people who attempt suicide will be 10 to 20 times greater [1]. Suicide is the leading cause of death among young people aged between 15 and 29 years [2]. In China, 200 million people attempt suicide annually, with two-thirds aged between 15 and 34 years [3]. Suicide prevention is thus crucial, particularly for young people.

Current suicide prevention methods practiced globally include school-based screening, screening by a primary care provider, and gatekeeper training, all of which are methods that are targeted at the general population and involve passively waiting for people to be in need [4]. However, many studies have found that because most people experiencing suicidal thoughts and behaviors tend not to participate in the aforementioned activities and have low motivation to seek help, existing methods have a rather weak effect on suicide prevention [5-8]. For example, only 17% of suicidal people in low-income countries, such as China, receive treatment in a timely manner [6]. The main reasons for not seeking help include the lack of a perceived need for services, high self-reliance, stigma, and structural factors such as time and cost [6-8]. However, the subjective judgment of at-risk individuals may not be good, and a high self-reliant tendency may lead to severe depressive symptoms and suicidal ideation among young people [9]. The passive suicide approach needs suicidal cases to actively seek help [10], such as the Columbia suicide screening program in which suicidal students filled in surveys in schools to get help [11]. In contrast, a proactive approach for suicide prevention, in which the program itself takes the initiative to identify suicidal people and invite them to use specific services, may increase the likelihood of service usage for the hidden people [10].

As in most developing countries, mental health care in China is at an early stage of development [12]. Moreover, because of China’s large population and uneven distribution of resources, it is hard to implement school-based screening or maintain primary care provider screenings nationally [13,14]. Gatekeeper training is still at an early stage [15], and thus new suicide prevention methods are urgently needed.

The internet has become an indispensable part of life for many people. As such, researchers have started to use people’s self-generated online messages to identify suicidal thoughts and behaviors either by manual [16] or machine learning analysis [17]. However, identification of suicidal thoughts and behaviors is just the first step in suicide prevention. Even though the internet has been used to manage suicide-prone crises [18,19], more effort is needed to prevent suicide. Previous studies have used the internet simply as a platform, and this means that they suffered from the same shortcomings as traditional prevention methods.

Half of the Chinese population uses the internet. Approximately two-fifths (40.9%) of China’s netizens use the Sina microblog, the Chinese version of Twitter [20]. Microbloggers (users of microblogs) can post microblog posts publicly, similar to Twitter. They can also send direct messages to other users that can be seen by the sender and receiver exclusively. Microbloggers can follow other users, along with replying to, commenting on, reposting, or liking others’ posts. An average of 139 million new posts are generated on the Sina microblog daily, with most (82%) microbloggers being under 30 years old [21]. These phenomena provide opportunities to prevent young people dying from suicide in China because existing findings suggest that young people feel they can freely discuss suicide-related topics on social networks [22,23]. The challenge is that the proportion of suicide posts is extremely low, making it nearly impossible to identify them manually, and that is why researchers have called for more efforts to build automated or semiautomated suicide ideation detectors to facilitate the provision of timely help and support to people at risk of suicide [24]. Suicidal people need to first be identified, and suicide identification and preventions can be carried out with the assistance of machine learning models.

To our knowledge, no research has previously been undertaken on the combination of proactive identification of suicidal individuals via social media and specialized crisis management. Moreover, a growing number of studies have demonstrated that (1) there is a shortage of online suicide prevention approaches [25], (2) unidirectional monologic suicide prevention information distributed by professional institutions is insufficient and more dialogic communication is needed between professionals and people at risk of suicide [26], (3) suicidal statuses can be detected from human language [27], and (4) a large improvement (such as enhancement of recall) can be achieved by applying machine learning algorithms to suicide identification [17]. Because language is an explicit behavior that indicates human mental status, people with suicide ideation are more likely to talk about suicide than people without suicide ideation [28]. Recently, researchers have started to use online longitudinal data to evaluate certain improvements after receiving psychosocial support services [29,30]. For example, people who used more future tense words online were found to have benefited more from online social support [31]. Previous studies have shown that higher future orientation was associated with less suicide ideation [32,33]. In our view, a reduction in death-oriented language and an increase in future-oriented language may serve as indicators for reduced suicide risk.

**Figure 1:**
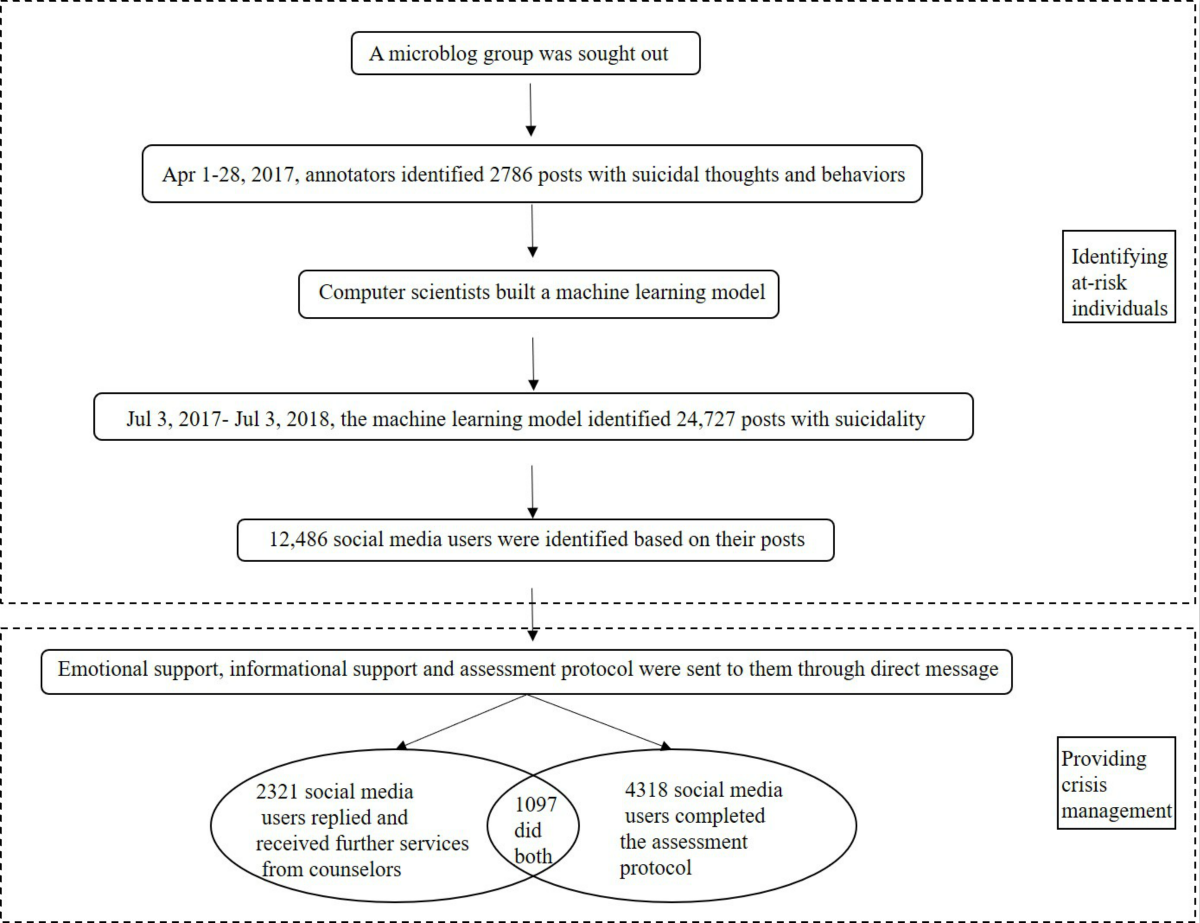
Study protocol for evaluating the Proactive Suicide Prevention Online (PSPO).

We proposed a new internet-based approach, Proactive Suicide Prevention Online (PSPO), for the identification and prevention of suicidal thoughts and behaviors (see Figure 1). We identified a microblog group online and manually annotated their comments on a suicide note to train a machine learning model. Next, the model was used to automatically identify posts that suggested suicidal thoughts and behaviors. We proactively provided crisis management in the form of emotional and informational support to microbloggers identified as at risk. Finally, we used the language changes in their posts as criteria to evaluate the efficacy of the PSPO approach. Based on the research gaps summarized above, we aimed to evaluate (1) the performance of PSPO in identifying high-risk social media users with suicidal thoughts and behaviors, (2) the acceptability of a proactive approach that offered help to social media users with suicidal thoughts and behaviors, (3) the improvement of PSPO in prompting suicidal social media users to seek help in comparison with traditional suicide prevention methods, and (4) the efficacy of PSPO for suicidal social media users in terms of changes in their language use (ie, reduced death-oriented words and increased future-oriented words).

## Methods

### Data Collection

#### Identifying At-Risk Individuals

A microblogger, Zoufan, died by suicide due to depression on March 17, 2012, with her suicide arousing wide attention online. Since her death, her blog has become a “secret garden” where suicidal people share their feelings and thoughts. By July 24, 2018, more than 1.3 million comments had been posted on her online suicide note, and many of them contained suicide information. This microblog group comprises people with and without suicide ideation. We analyzed the comments left on Zoufan’s online suicide note. The official Sina microblog application programming interface was used to obtain comments posted from April 1 to 28, 2017, which were manually annotated as the training set. Comments posted from July 3, 2017, to July 3, 2018, were obtained and automatically identified by the developed machine learning model. Further details regarding the procedures used for building the machine learning model (eg, the coding system for suicidal thoughts and behaviors in posts, feature selection for the machine learning models) are provided in Data Analysis.

#### Providing Crisis Management

All microbloggers who were identified by the machine learning model as expressing suicidal thoughts and behaviors were invited to join the study via direct message. There were no exclusion criteria because we aimed to reach out and provide support to as many suicidal social media users as possible.

The direct message, designed in our previous study [34], included (1) a brief introduction to the project; (2) URLs for assessment protocols on suicidal thoughts and behaviors, depressive symptoms, and help-seeking behaviors; (3) emotional support (empathy, recommendations such as having regular physical exercise and a healthy diet, and encouragement); (4) informational support (the URL for this study, along with referrals to hospitals and hotline services); and (5) details regarding the availability of one-to-one counseling by contacting counselors via direct messaging (see Multimedia Appendix 1).

If a user replied to the direct message, counselors provided support that was targeted to the user’s specific problem. Twelve certified counselors (2 men and 10 women, mean age 23.08 [SD 1.08] years) with experience in handling suicidal cases were trained to provide counseling services through direct messaging.

#### Direct Message Assessment Protocol

Suicidal thoughts and behaviors were tested with the use of two items chosen based on previous research [35] and the 9-Item Patient Health Questionnaire (PHQ-9) [36]. The two items were “Do you have a plan to commit suicide?” and “Have you ever attempted suicide?” Participants responded with binary choices (yes/no), and if the answer was yes to the first item, they were required to indicate whether they had a specific or vague plan. A sample item on the PHQ-9 would be “having little interest or pleasure in doing things.” Participants rated the frequency of the 9 symptoms over the past 2 weeks on a 4-point Likert scale (0 = not at all, 3 = nearly every day), and the total score of the PHQ-9 ranges from 0 to 27, with higher scores indicating a greater severity of depressive symptoms. The Chinese version has been shown to have good psychometric properties [37], and internal consistency was .84 in this study.

Help-seeking behavior was assessed by two items [35]: “What kind of psychological treatment have you received before?” and “Have you sought help when you had suicidal ideation?” and if the answer was positive to either of the questions, the effectiveness of the former help was rated on a 7-point Likert scale (1 = totally disagree, 7 = totally agree). If the participant’s rating for the question was 3 or lower, we recorded that the former help was not useful.

Acceptability was measured with one item (“How acceptable do you find this proactive help?”) and rated on a 7-point Likert scale (1 = totally disagree, 7 = totally agree). We considered a rating of 4 or higher to indicate that the program was acceptable.

#### One-to-One Counseling

The counselor training was based on problem-solving therapy [38], which begins with identifying a person’s problem and then helping them to affirm feasible solutions to a specific issue by setting goals and comparing the pros and cons of every plausible solution. A concrete and feasible plan is then made to facilitate the client in overcoming the problem they face. The goal for counselors in crisis management was to persuade suicidal microbloggers to seek professional services and provide them with the appropriate referrals. The interaction between the microbloggers and our counselors also depended on the needs of the microbloggers. The training lasted 3 hours and included a theoretical explanation and practice of applying problem-solving therapy to this online situation. In addition to counselors being under monthly supervision by psychiatrists and senior counselors, we also formed an online chat group where the counselors could discuss the problems they encountered in consultations at any time.

Because the data used in this study were all publicly available, traditional informed consent was not appropriate. In the identification section, measures were taken to anonymize the data in the data analysis to minimize the inadvertent disclosure of personal information or information that may reveal clues with regard to an individual’s online identity. In the crisis management part of the study, participants gave informed consent voluntarily when agreeing to take part in the program. The project received ethical approval from the Institutional Review Board of the Institute of Psychology, Chinese Academy of Sciences, with the ethics approval number H16003.

### Data Analysis

#### Building Machine Learning Models for Suicide Recognition

The first step for supervised learning was to obtain a training set. To achieve good performance of the machine learning model, this study decided not to use crowd-sourcing [39]. Rather, 5 psychology postgraduates with expertise in analyzing suicide annotated the microbloggers’ comments. The annotation process was identical to the one used in our previous study [34]. Expressing a death wish or writing about suicide was coded as suicide ideation. Because a suicide plan is defined as suicide-related communication to account for its interpersonal nature, which is often expressed in verbal words regarding how one might advance from ideation to action [40], we operationalized a suicide plan as one that involved discussions regarding the act of dying (eg, a death kit, death place and time, making a will) after considering the nature of dialogue in social media. Attempted suicidal behavior within the preceding 2 weeks with current suicide ideation or the possibility of executing a suicide plan in the coming 1 to 2 weeks was coded as a suicide attempt. Posts were ranked as follows: 0 = no suicide risk; 1 = risk of suicide ideation but no detailed plan made; 2 = risk of suicide plan not requiring emergency aid; and 3 = significant risk of suicide attempt requiring emergency aid.

Comments identified as indicating suicidal thoughts and behaviors were labeled as positive training samples, and 10,000 posts without suicidal thoughts and behaviors were randomly selected to serve as negative training samples. Another strategy for the improvement of the performance of the machine learning model was the theoretical-based feature selection. Researchers in the field of computer science had a tendency to select predictive features by randomly using linguistic analyses, such as n-grams and sentiment analyses, without a theoretical or empirical basis [41,42]. In this study, we combined data-driven features (n-grams) that were derived from social media data, domain knowledge, and theoretical guidance [43,44] to select features. A knowledge-based generic suicide-related lexicon [45], which was manually developed by a panel of domain experts, was used. Theory-motivated features include personality and depression, which are the factors most commonly cited as being relevant to suicides [46-48]. Personal traits such as fearfulness, social inhibition, shyness, pessimism, immaturity, and lack of internal organization were associated with psychotic suicide attempts [43]. Moreover, there has been significant progress in predicting personality and depression from social media data [43,44,49,50], and depression has also been used to predict suicidal behaviors [51]. Thus, it is both theoretically sound and technically feasible to incorporate predictive features of personality traits and depression into a model for suicide ideation detection. To our knowledge, no prior studies have used the lexicon and predictive features (including personality traits and depression) that were included in the machine learning models.

A binary classification of suicidal thoughts and behaviors detection model was built to determine if the comment indicated suicidal thoughts and behaviors. We used support vector machine (SVM), decision tree, random forest, and logistic regression algorithms with 10-fold cross-validation to train the detection model because these machine learning algorithms are the most widely used methods of predicting psychological characteristics and emotions and detecting suicide ideation [45,52]. The performance of the detection model was evaluated through the use of four metrics: precision, recall, F-measure, and accuracy [53].

#### Using Language Frequency Changes as an Efficacy Indicator for Crisis Management

Because of the low response rate in the completion of the online survey, we were unable to collect 1-month post–PHQ-9 data. We used language frequency changes as an efficacy indicator for crisis management. To examine the microbloggers’ language changes between 1 month before the commencement of the program and 1 month after the completion of the program, the Simplified Chinese Linguistic Inquiry and Word Count (SCLIWC) was used. The SCLIWC is an amended version of the text analysis program LIWC designed to perform well in Simplified Chinese on a microblog [54]. It is composed of 7 main categories and 64 subcategories. Death-oriented and future-oriented words are 2 of the subcategories, and every participant’s posts were parsed into these 2 subcategories. Category scores were calculated by the ratio of words within the category to all the words in the posts. Furthermore, we used the change tendencies of the 2 subcategories as a measure of the efficacy of the program.

## Results

### Machine Learning Models for Suicide Recognition

From April 1 to 28, 2017, four weekly sessions of manual annotation of comments were conducted. Of the 27,007 comments, 10.32% (2786/27,007) were identified as indicating suicidal thoughts and behaviors. In those, 81.44% (2269/2786), 13.75% (383/2786), and 4.81% (134/2786) contained information coded as suicide ideation, suicide plan, and suicide attempt, respectively (Table 1).

Table 2 presents the means and standard deviations in the performances of the detection models with the whole feature set and two baseline feature sets developed by the selected classification algorithms. The best overall models were the SVM models. We compared the performances of SVM models, which were constructed with each feature set using a Tukey honestly significant difference post hoc test. The precision of the model of set C is lower than set A (*t*=–6.32, *P*<.001). However, the recall (*t*=12.07, *P*<.001), F-measure (*t*=5.48, *P*<.001), and accuracy (*t*=3.32, *P*=.004) of the model were all significantly higher in set C than with set A. Comparing the performance of SVM models using the feature sets C and B, and despite the precision of the SVM model with set C also being lower (*t*=–5.80, *P*<.001), the recall and F-measure of the model using set C were significantly higher (*t*=12.23, *P*<.001 and *t*=3.87, *P*=.001, respectively) than those of the model using set B, while their accuracies were equivalent (*t*=1.34, *P*=.20).

Of the 387,823 comments made between July 3, 2017, and July 3, 2018, 24,727 (6.38%) were identified as being indicative of suicidal thoughts and behaviors by the machine learning model.

**Table 1:** Manual identification of suicidal comments posted in April 2017.

Date	Comments (n)	Suicidal comments			
		Suicidal thoughts and behaviors, n (%)	Suicide ideation, n (%)	Suicide plan, n (%)	Suicide attempt, n (%)
4/01-4/07	6975	849 (12.17)	702 (82.69)	107 (12.60)	40 (4.71)
4/08-4/14	6201	682 (11.00)	561 (82.26)	90 (13.20)	31 (4.55)
4/15-4/21	6467	563 (8.71)	457 (81.17)	82 (14.56)	24 (4.26)
4/22-4/28	7364	692 (9.40)	549 (79.33)	104 (15.03)	39 (5.64)
Total	27,007	2786 (10.32)	2269 (81.44)	383 (13.75)	134 (4.81)

**Table 2:** Performance of the machine learning models.

Model performance and feature set		SVM^a^, mean (SD)	DT^b^, mean (SD)	RF^c^, mean (SD)	LR^d^, mean (SD)
**Precision**					
	A^e^	.88 (.01)	.84 (.02)	.87 (.01)	.87 (.01)
	B^f^	.88 (.01)	.76 (.01)	.85 (.01)	.87 (.01)
	C^g^	.85 (.01)	.76 (.01)	.85 (.01)	.88 (.01)
**Recall**					
	A	.78 (.02)	.68 (.05)	.75 (.02)	.79 (.02)
	B	.80 (.01)	.75 (.01)	.74 (.01)	.79 (.01)
	C	.85 (.01)	.75 (.01)	.73 (.01)	.80 (.01)
**F-measure**					
	A	.83 (.01)	.74 (.03)	.80 (.01)	.83 (.01)
	B	.84 (.01)	.76 (.01)	.79 (.01)	.83 (.01)
	C	.85 (.01)	.76 (.01)	.78 (.01)	.84 (.01)
**Accuracy**					
	A	.85 (.01)	.79 (.02)	.83 (.01)	.85 (.01)
	B	.86 (.01)	.78 (.01)	.82 (.01)	.85 (.01)
	C	.86 (.01)	.78 (.01)	.82 (.01)	.86 (.01)

^a^SVM: support vector machine.

^b^DT: decision tree.

^c^RF: random forest.

^d^LR: logistic regression.

^e^A: n-gram features.

^f^B: n-gram features + domain knowledge features.

^g^C: n-gram features + domain knowledge features + theory-motivated features.

### Crisis Management

We sent direct messages to the 12,486 microbloggers (some microbloggers had multiple comments) who were identified as having expressed suicidal thoughts and behaviors in the 24,727 comments by the machine learning model. A total of 34.58% (4318/24,727) of individuals completed the assessment protocol, and there were 1403 valid samples (mean age 21.66 [SD 3.26] years). Females significantly outnumbered males (χ^2^_1_=647.33, *P*<.001), and most of the participants were students or employed, single, and had graduated from college (see Table 3).

In terms of suicide risk, most of the respondents (1259/1403, 89.73%) thought they would be better off dead or by hurting themselves in some way. Nearly half of these (699/1403, 49.82%) had a suicide plan, with 6.34% (89/1403) indicating a specific plan to commit suicide and 43.48% (610/1403) indicating a vague plan to do so. Of the 1403 participants, 545 (38.85%) had previously attempted suicide. The mean score for the PHQ-9 was 17.40 (SD 5.98), and this represents moderately severe depressive symptoms. Two-thirds of the participants (924/1403, 65.86%) had never received any kind of psychological treatment. Just over half (725/1403, 51.67%) had not sought help from anyone, 12.19% (171/1403) had sought help from professionals (such as a psychiatrist, therapist, or general practitioner), and 36.14% (507/1403) had sought help from people around them (such as family and friends). Of the participants who had sought help before, 48.33% (678/1403) rated the efficacy of the former help as 2.60 (SD 1.43), and 77.00% (522/678) thought that the former help rendered was of no use to them.

On a 7-point Likert scale, nearly 70% (968/1403, 69.00%) of participants rated proactive help through the use of direct messaging acceptable (4 or more on the scale). The average score for all participants was 4.35 (SD 1.81).

**Figure 2:**
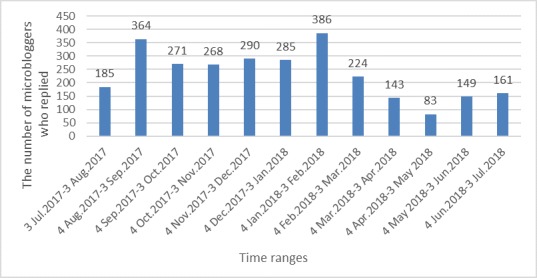
Number of microbloggers who interacted with our counselors from July 3, 2017, to July 3, 2018.

Between July 3, 2017, and July 3, 2018, microbloggers logged into the study website to view prevention information 12,300 times. A total of 2321 users replied to a direct message at least once. Figure 2 shows the total number of microbloggers who interacted with our counselors monthly from July 3, 2017, to July 3, 2018. On average, there were 234.08 (SD 88.70) microbloggers who interacted with our counselors every month. Table 4 shows the interaction between microbloggers and counselors, and this included the number of microblogger replies and interaction days. Approximately 90% (2043/2321, 90.12%) of the microbloggers replied fewer than 10 times. Nearly 97% (2246/2321, 96.77%) of the microbloggers interacted with our counselors for less than 5 days. A total of 1097 users completed an assessment protocol and consultation. Of the 12,486 microbloggers who were contacted, 5542 (44.39%) responded to our direct message either by completing the assessment protocol or by interacting with the counselors. Earlier studies found that the percentages of college students seeking help from professionals were 5.1% for all college students, 14.4% for college students with mental health problems, and 4.5% for college students without mental health problems [55]. Compared with the traditional methods used, we were able to prompt a larger number of people identified via the machine learning model as having posted suicidal content to seek help for their distress or suicide ideation.

**Table 3:** Demographic characteristics of participants.

Characteristic		Value, n (%)
**Gender**		
	Male	225 (16.04)
	Female	1178 (83.96)
**Education level**		
	Junior middle school or below	82 (5.84)
	Senior high school	272 (19.39)
	College	1006 (71.71)
	Graduate or above	43 (3.06)
**Employment status**		
	Employed	472 (33.64)
	Unemployed	229 (16.32)
	Student	603 (42.98)
**Marital status**		
	Single/divorced	1342 (95.65)
	Married	61 (4.35)

**Table 4:** Interactions between microbloggers and counselors.

Interactions		Value, n (%)
**Number of microblogger replies**		
	≤10	2043 (90.12)
	11-30	118 (5.21)
	31-50	46 (2.03)
	51-100	60 (2.65)
	>100	54 (2.38)
**Days of interactions with counselors**		
	≤5	2246 (96.77)
	6-10	48 (2.07)
	>10	27 (1.16)

**Table 5:** Changes in frequency of language use pre- and postprogram.

Category	Examples	Preprogram, % (SD)	Postprogram, % (SD)	*t* value	*P* value
Death-oriented words	Die/suicide/will	0.37 (0.01)	0.31 (0.01)	2.21	.03
Future-oriented words	After/soon/future	0.34 (0.01)	0.34 (0.01)	–2.29	.02

Finally, we used SCLIWC to detect language changes in the 2321 social media users who replied to the direct message. By tracing the accounts of those microbloggers, we compared their microblog posts from a month before and after receiving services from our counselors. After deleting users who did not complete the interaction with the counselors for one month, there were 2031 microbloggers. As shown in Table 5, the frequency of death words significantly declined (*P*=.03), and the frequency of future-oriented words significantly increased (*P*=.02). For the month before the program, the number of posts by a single user ranged from 1 to 1013. The mean was 30.59 (SD 84.36). For the month after the program, the number of posts by a single user ranged from 1 to 1279, and the mean was 27.41 (SD 74.04). The paired sample *t* test result showed that the difference in the total number of posts by a single user before and after the program was thus not significant (*t*=1.92, *P*=.06).

## Discussion

### Principal Findings

In our study, we first identified a microblog group formed around the Sina microblog account of a microblogger who committed suicide, which was an efficient way to identify a high-risk population. Then, we proactively pushed direct messages to invite all of the microbloggers identified by the machine learning model as people who had exhibited suicidal thoughts and behaviors to participate in our study. Our results provided some preliminary evidence that automatic identification of suicidal thoughts and behaviors along with proactive suicide prevention are acceptable and helpful.

For suicide ideation detection, recall is arguably more important than precision. The results of machine learning models demonstrate that in general, incorporating theory-related features and features based on domain knowledge can improve the recall, F-measure, and accuracy of a model for detecting suicide ideation. The best results were .88, .85, .85, and .86 for precision, recall, F-measures, and accuracy, respectively, which demonstrates the utility of the model in identifying suicide posts. Apart from multiresources and theoretical-based feature selection [39,41] being able to ensure that relevant features are included and redundant ones excluded, our results outperformed earlier suicide machine learning models to identify posts with suicide content [52,56] mainly because we did not rely on crowd-sourcing [39] and instead opted for postgraduate annotators specializing in studying suicide. In addition, the dataset size was almost twice the size of those used in similar studies done earlier [56,57].

In this study, most individuals who completed the questionnaires were single females (employed or students) with a college degree. This is consistent with a previous study showing that females are more likely to talk about suicide ideation to health professionals and use health services than males [6]. Those with a higher education and those who are never married also had significantly higher odds of receiving mental health treatment [6].

For the period of July 3, 2017, to July 3, 2018, 6.38% of the comments were identified as expressive of suicidal thoughts and behaviors. The self-reports of the 1403 participants identified as individuals who displayed suicidal proclivities testified to the utility of the machine learning model. The self-reported results in the survey showed that percentages for suicide ideation, suicide plan, and past suicide attempt were 89.73%, 49.82%, and 38.85%, respectively. These are much higher than the results in a meta-analysis study that showed percentages of suicide ideation and past suicide attempt for the general Chinese population were 3.9% and 0.8%, respectively [58]. Moreover, 6.34% of our participants self-reported a specific suicide plan. Their mean score for the PHQ-9 was 17.40 (SD 5.98), indicating moderately severe depressive symptoms. If suicide ideation can be identified as early as possible, then at-risk individuals can be prevented from deteriorating to a point where they make a specific suicide plan [59]. PSPO enables timely crisis management only for those in need, without disturbing others.

Even with a high suicide ideation rate, 65.86% of participants who completed the questionnaires had never received any kind of psychological treatment. Moreover, 51.67% of them did not seek help from anyone regarding their suicide problem. This is consistent with earlier studies [60,61] and may explain why 69.00% of the participants accepted our PSPO—it provided a new way of accessing help for those who had experienced barriers to seeking help for suicidal thoughts and behaviors previously. Another possible reason for the acceptability of PSPO was its anonymous nature.

This study has demonstrated some primary evidence for the efficacy of PSPO. Eliminating suicide ideation can be a long process, but suicide crisis management can serve as “emotional cardiopulmonary resuscitation” for people at risk. We sent out 24,727 direct messages to 12,486 different social media users, and 5542 (44.39%) of them responded. Of these, 4318 individuals finished the assessment protocol and 2321 users replied to the direct message. On average, 234.08 (SD 88.70) microbloggers interacted with our counselors monthly, approximately 90% of the microbloggers replied fewer than 10 times, and nearly 97% of the microbloggers interacted with our counselors for less than 5 days. Those results indicate that PSPO might largely extend the potential for suicide prevention for those who have never sought help before when compared with traditional passive methods. Moreover, the prevention information on our website was viewed 12,300 times. Finally, after interacting with the counselors, the microbloggers with suicidal thoughts seemed to change the language they used on social media significantly. In particular, the frequency of death-oriented words was found to have been significantly reduced a month after the crisis management as compared with the frequency of those words one month prior to receiving crisis management. One possible reason could be that the microbloggers felt the concern, social support, and empathy of the counselors. Another possible reason is that these users started to seek help after the consultation. At the same time, the frequency of future-oriented words increased significantly, although it was a slight change. This may be due to the relatively small number of future words in the overall vocabulary used. Nevertheless, it may also signal that the users had less suicide ideation and became more willing to accept support than before.

### Limitations and Future Work

In this study, we only focused on a microblog group. Future studies would be needed to establish whether our machine learning model can be applied to other similar suicide groups and other social media platforms such as school bulletin boards, suicide groups online, or online self-help groups for suicidal thoughts and behaviors. Because our suicidal thoughts and behaviors detection model is at an early stage of development, only a binary classification model was built, and it was mainly focused on finding suicidal candidates for the primary crisis management. Multiclass classification can be adopted and adapted in the future to facilitate customized suicide prevention for different social media users. Moreover, to detect suicide ideation, we concentrated mainly on text features extracted from posts, although other behaviors on social media, such as interactions with other users and posting frequencies and times, could also be effective predictors. Examining these potential factors may provide additional insights and guidance for building more effective models based on social media to detect suicide ideation.

Slightly more than one-third (34.58%) of the users completed the questionnaires provided in the direct message. Given the sensitivity of the subject, the relatively low response rate is understandable, although it is higher than those found in earlier studies (close to 10%) [62]. For future research, we plan to investigate the differences between those who participated in the program and those who did not to acquire more first-hand data to develop a deeper understanding of their psychology and behavior. Our aim is to involve more human resources in lifesaving, thereby increasing the prevention rate for suicide.

We only offered the primary crisis management information to microbloggers with suicidal thoughts and behaviors. More standardized and systematic emergency intervention protocols, mental health resources, and professional referrals are needed to guarantee reasonable retention in a future study. While PSPO provides an opportunity for longitudinal study, the effectiveness of various Web-based suicide prevention and intervention approaches including PSPO should be examined because a follow-up is crucial in suicide intervention [63]. We will thus try our best to provide follow-up measures and actions for the identified users.

Finally, because this was a preliminary study, we mainly used the changes in future-oriented word frequency to demonstrate the efficacy of PSPO. Our efficacy evidence should be interpreted with caution because the relationship between future-oriented words and reduced suicide risk would still need further verification. Future studies should consider using direct indicators of reduction of suicidal thoughts and behaviors to demonstrate the improvement. Moreover, there is a possibility that the results reflect regression to the mean. A few strategies in the study design stage (eg, using a randomized controlled trial, having multiple tests at different time points for actual behavior instead of just intention or attitude) and analysis stage (eg, using analysis of covariance) are desirable to reduce the regression to the mean [64,65].

### Conclusion

This paper presents PSPO as a proactive suicide prevention method for identifying and preventing suicide incidents by social media users, especially young people. The results indicate that PSPO is feasible for identifying populations at risk of suicide and providing effective crisis management, and the identification of at-risk individuals is automatic and timely. The crisis management is also proactive, acceptable, and low cost. Our study may be a useful supplement to existing prevention programs, and suicide crisis management may increase public awareness of help-seeking related to suicide risk, thereby improving the well-being of the population. This approach could alleviate the problems associated with a huge population with weak psychological services and help with an imperfect suicide prevention system in large developing countries such as China.

## Appendix

Multimedia Appendix 1 The direct message sent to users with suicidal thoughts and behaviors.

## Abbreviations

DT: decision tree
PSPO: Proactive Suicide Prevention Online
SCLIWC: Simplified Chinese Linguistic Inquiry and Word Count
PHQ-9: 9-Item Patient Health Questionnaire
RF: random forest
SVM: support vector machine
