# Supplementary material for: Proactive Suicide Prevention Online (PSPO): Machine Identification and Crisis Management for Chinese Social Media Users With Suicidal Thoughts and Behaviors
Source: J Med Internet Res. 2019 May 8;21(5):e11705. doi: 10.2196/11705 (PMC6682269; doi:10.2196/11705)
Supplement: Multimedia Appendix 1 [file jmir_v21i5e11705_app1.pdf]

Hello, we are a volunteer counsellor team organized by the Beijing Social Work Committee and the Institute of Psychology, Chinese Academy of Sciences. We saw your comments under Zoufan's post. Are you okay now? How are your feelings and emotions now? If you need help, you can log onto the following website: http://ccpl.psych.ac.cn/PsyMap/quiz/index/. There, you can get a free psychological assessment and other psychological assistance resources (such as the Beijing Psychological Crisis Intervention Centre 24-hour free hotline: 010-82951332). Our counsellors will give you feedback based on your assessment results, and you may receive further psychological assistance if your condition is severe. Our counsellors provide online counselling services from 18:00 to 22:00 every day. If you are having a difficult time, there are many people who care about you and wish you well.
